# Sociodemographic Patterns of Exclusive and Dual Combustible Tobacco and E-Cigarette Use among US Adolescents—A Nationally Representative Study (2017–2020)

**DOI:** 10.3390/ijerph19052965

**Published:** 2022-03-03

**Authors:** Bukola Usidame, Jana L. Hirschtick, Delvon T. Mattingly, Akash Patel, Megan E. Patrick, Nancy L. Fleischer

**Affiliations:** 1 School of Public Health, University of Michigan, Ann Arbor, MI 48109, USA; janahirs@umich.edu (J.L.H.); delvon@umich.edu (D.T.M.); akapatel@umich.edu (A.P.); nancyfl@umich.edu (N.L.F.); 2 Institute for Social Research, University of Michigan, Ann Arbor, MI 48106, USA; meganpat@umich.edu

**Keywords:** nicotine, tobacco, cigarettes, e-cigarettes, dual use, adolescents, youth, disparities

## Abstract

This study assessed the sociodemographic predictors of exclusive and dual use of the most frequently used nicotine/tobacco products, e-cigarettes, and combustible tobacco among adolescents. Cross-sectional data was from the 2017–2020 Monitoring the Future nationally representative study of eighth, tenth, and twelfth-grade students. We coded past 30 day nicotine/tobacco use into four mutually exclusive categories: no use, e-cigarette use only, combustible use (cigarette or cigar) only, and dual use (e-cigarette and combustible). We pooled the 2017–2020 data to examine the relationship between sex, race/ethnicity, parental education, and each product-use category using multinomial logistic regression, stratified by grade level. Among eighth (*N* = 11,189), tenth (*N* = 12,882), and twelfth graders (*N* = 11,385), exclusive e-cigarette use was the most prevalent pattern (6.4%, 13.2%, 13.8%, respectively), followed by dual use (2.7%, 4.5%, 8.9%), and exclusive combustible use (1.5%, 2.5%, 5.3%). eighth and tenth-grade adolescents whose highest parental education was a 4-year college degree or more had lower odds of exclusive combustible and dual use when compared to adolescents whose highest parental education was less than a high school degree. Research should continue to monitor the differential use of combustible tobacco products and e-cigarettes among adolescents from low socioeconomic status backgrounds or racial/ethnic minority households to inform ongoing and future interventions or policies.

## 1. Introduction

Tobacco or nicotine product use among adolescents is a major public health crisis in the United States, specifically with the rapid increase in e-cigarette use in recent years [1,2,3,4,5]. E-cigarettes are the most commonly used nicotine products among adolescents, followed by cigarettes and cigars [4,6]. Among eighth, tenth, and twelfth graders, past 30 day cigarette use ranged from 1.9% to 9.7%, past 30 day cigar use ranged from 1.3% to 10.1%, and past 30 day e-cigarette use ranged from 3.5% to 25.5% from 2017 to 2020 [6]. Furthermore, dual use of any two nicotine/tobacco products ranged from 2.4% to 4.0% among middle school students and from 8.2% to 10.8% among high school students from 2017 to 2020, with cigarettes, cigars, and e-cigarettes being the most frequently combined products [2,3,4]. Research suggests that e-cigarette use may increase the risk of dual nicotine/tobacco use among adolescents [6], which has been linked with increased school- and substance-related risk behaviors [7,8].

Although e-cigarettes are currently the predominant nicotine product used by adolescents, studies have highlighted the need to continue to assess combustible products (cigarettes or cigars) use because of their health impacts. Individuals who use combustible tobacco products face health consequences such as cancers, though those who smoke cigars are at a higher risk of oral cancer, with increased frequency of use and extent of inhalation than those who smoke cigarettes [9]. Nicotine content and delivery differs between cigarettes and cigars (cigars generally deliver more), but both products contain enough nicotine to eventually cause addiction [10,11]. Dual use of e-cigarettes and combustible tobacco products has potential health risks and increases the tendency for nicotine addiction relative to single product use [11]. Hence, there is a need to monitor the use of e-cigarettes and combustible products to help public health officials understand the potential adverse impact of these products among adolescents.

Studies have shown slight sociodemographic differences among adolescents who exclusively use e-cigarettes versus combustible tobacco products. While male adolescents are more likely than females to use e-cigarettes exclusively [12,13], studies have shown comparable rates of exclusive combustible tobacco product use among males and females [4,12]. Unlike exclusive e-cigarette use, which is more common among non-Hispanic White adolescents compared to racial/ethnic minority adolescents, non-Hispanic Black adolescents are more likely to use combustible tobacco [2,4] when compared to non-Hispanic White adolescents. Adolescents who use e-cigarettes exclusively are also more likely to have a higher socioeconomic status (SES), while adolescents who exclusively use combustible tobacco products are more likely to have a lower SES. On the other hand, there are sociodemographic similarities among adolescents who use e-cigarettes exclusively and those who use e-cigarettes and combustible tobacco products (dual use) [12,13]. These adolescents are more likely to be male than female, non-Hispanic White than a racial/ethnic minority, and have more highly-educated parents than less-educated parents [12,13].

Our study aims to build upon the current literature by examining sociodemographic differences in exclusive and dual use of e-cigarettes and combustible tobacco products (cigarettes and cigars combined) among adolescents using data from 2017–2020. There has been a great deal of focus on non-combustible nicotine use, but combustible use remains a concern with 13.4% of eighth, tenth, and twelfth graders using cigarettes and/or cigars in 2019—the second and third most commonly used tobacco products, respectively, among adolescents [5,6]. Recognizing the potential health risks associated with combustible and non-combustible product use, it is particularly important to examine adolescent exclusive and dual use of these products. In addition, prevalence estimates of nicotine/tobacco product use have not commonly considered the exclusive use of these products [5,6,14,15]. While any use is a vital measure of tobacco use, evidence has shown that distinguishing exclusive use from multiple product use is essential to identify associated risk factors and targeted intervention [16]. Using a nationally representative study, we assessed patterns of exclusive e-cigarette use, exclusive combustible tobacco product use, and dual use of e-cigarettes and combustible tobacco among eighth, tenth, and twelfth graders overall and by sex, race/ethnicity, and parental education.

## 2. Materials and Methods

### 2.1. Data Source

We used data from 2017 to 2020 from the Monitoring the Future (MTF) study, a nationally representative cross-sectional sample of eighth, tenth, and twelfth-grade students [15]. Data collection takes place annually from over 40,000 students in approximately 400 public and private schools throughout the United States. About 14,000 eighth grade students (mostly in middle schools), 15,000 tenth grade students, and 13,000 twelfth grade students are surveyed each year. Though, the 2020 sample size was about 25% of the typical data collection as a result of the data collection being suspended due to the pandemic in March 2020 [15]. A random sampling procedure is used for sample collection in three stages: geographic areas, schools within the areas, and students within the schools. Weights are assigned to compensate for differential probabilities of selection at each stage of sampling. eighth and tenth-grade students are randomly assigned to one of four survey forms, while twelfth-grade students are randomly assigned to one of six survey forms. Questions asking individual respondents about cigar and e-cigarette use were included on three of the forms for eighth, tenth, and twelfth graders, while a question on cigarette use was asked on all forms for all grades from 2017 to 2020 (Supplemental Table S1). Surveys were administered via paper and pencil in 2017–2018, in 2019 half of participants responded via paper and half via electronic tablets, and in 2020 all respondents used tablets [17].

### 2.2. Nicotine/Tobacco Use Variable

Current use was defined as any use of a particular product in the past 30 days. To assess cigarette use, respondents were asked, “*How frequently have you smoked cigarettes during the past 30 days?*” Current cigarette use was coded as 0 for respondents who selected ‘Not at all,’ while all other use was coded as 1. The cigarette question was consistent across all the selected forms from 2017 to 2020 (Supplemental Table S1). Cigar use questions were consistently asked on certain forms (Supplemental Table S1). Respondents were asked, “*During the LAST 30 DAYS, on how many days (if any) have you: a. smoked large cigars? b. smoked flavored little cigars or cigarillos? c. smoked regular little cigars or cigarillos?*” Respondents who selected ‘None’ were coded as 0, while those who selected one or more days for at least one cigar product were coded as 1 to represent current cigar use.

E-cigarette use questions were asked in three different ways across the forms (Supplemental Table S1). Some respondents were asked, “*During the LAST 30 DAYS, on how many days (if any) have you…vaped an e-liquid with nicotine?*” Another group of respondents was asked, “*During the LAST 30 DAYS, on how many days (if any) have you used an electronic vaporizer such as an e-cigarette??*” Then “*On how many occasions (if any) have you vaped NICOTINE…during the last 30 days?*” The final group of respondents was asked, “*On how many days (if any) have you vaped NICOTINE…during the last 30 days?*” Across all e-cigarette questions, respondents who selected ‘None’ were coded as 0 while those who selected 1 or more days/occasions were coded as 1 for current e-cigarette use. We pooled all three questions together as one e-cigarette measure similar to previous studies [18,19].

We coded current tobacco use into four mutually exclusive categories: (1) no use of cigarettes, cigars, or e-cigarettes, (2) exclusive combustible tobacco product use (cigarette or cigar), (3) exclusive e-cigarette use (vaping nicotine) and (4) dual combustible and e-cigarette use. 

### 2.3. Sociodemographic Variables

Sociodemographic variables included sex (male or female), race/ethnicity (Hispanic, Non-Hispanic White (NHW), Non-Hispanic Black (NHB), or Non-Hispanic Other (includes all other races not previously mentioned that do not identify as Hispanic/Latino) or multiracial (NHO)), and parental education. Parental education was included as a proxy for socioeconomic status [20] and defined as the highest educational level of either mother or father (High school degree (HS) or less, Some college, or a 4-year college degree or higher).

### 2.4. Statistical Analyses

We pooled data from 2017–2020 to calculate weighted descriptive statistics for all study variables by grade and to conduct multinomial regression models. We ran crude and adjusted multinomial regression models to assess the relationship between sociodemographic factors (sex, race/ethnicity, and parental education) and the four-category nicotine/tobacco product use variable (referent group: no use). Each adjusted model controlled for year, to account for changes in patterns of use that might have occurred from 2017 to 2020, and sex, race/ethnicity, and parental education. All analyses were stratified by grade level: eighth grade, tenth grade, and twelfth grade, and models were adjusted for multiple testing using the Bonferroni correction method. Statistical analyses were conducted using Stata 15 (StataCorp, College Station, TX USA).

## 3. Results

### 3.1. Descriptive Statistics

**Figure 1:**
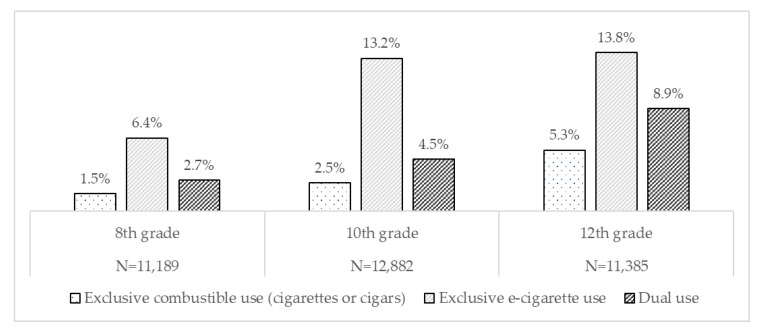
Prevalence of exclusive and dual use of combustibles (cigarette and cigars) and e-cigarettes among eighth, tenth, and twelfth graders: Monitoring the Future Surveys, 2017 to 2020.

The overall study sample for each grade from 2017 to 2020 was similar across grades; 11,189 for eighth grade, 12,882 for tenth grade, and 11,385 for twelfth grade (Table 1). The weighted distribution for each sociodemographic characteristic is included in Table 1. Female participants were 52.6% and NHW participants were approximately half (49.8%, 51.5%, and 54.0%) of the sampled population across the eighth, tenth, and twelfth grades, respectively. More than half of the participants had the highest parental education of a college degree or higher, with 60.8% in eighth grade, 58.4% in tenth grade, and 52.2% in twelfth grade. Among eighth, tenth and twelfth graders, respectively, exclusive e-cigarette use was most prevalent (6.4%, 13.2%, 13.8%), followed by dual use (2.7%, 4.5%, 8.9%) and exclusive combustible use (1.5%, 2.5%, 5.3%) for 2017 to 2020 (Table 1, Figure 1).

### 3.2. Regression Models

Tobacco use patterns varied by sex and grade (Table 2, Table 3 and Table 4). Males had lower odds than females of exclusive e-cigarette use in eighth grade, higher odds of dual use in tenth and twelfth grade, and higher odds of exclusive combustible use in twelfth grade, compared to no use. Male eighth graders had 19% lower odds than females of using e-cigarettes exclusively when compared to no use (aOR = 0.81, 95% CI = 0.68–0.98) (Table 2). However, male adolescents had higher odds than females of dual use in tenth (aOR = 1.67, 95% CI = 1.33–2.09; Table 3) and twelfth (aOR = 2.16, 95% CI = 1.82–2.57; Table 4) grades when compared to no use. In twelfth grade, males had higher odds than females of exclusive combustible use (aOR = 1.49, 95% CI = 1.21–1.84). We found no association between sex and exclusive combustible use in eighth and tenth grades, sex and exclusive e-cigarette use in tenth and twelfth grades, and sex and dual use in eighth and twelfth grades. 

By race/ethnicity, adolescents from racial/ethnic minority groups generally had lower odds of exclusive e-cigarette and dual use when compared to NHW adolescents, with mixed results on the association between race/ethnicity and exclusive combustible use (Table 2, Table 3 and Table 4). In all the adjusted models, racial/ethnic minority adolescents had lower odds than NHWs of using e-cigarettes exclusively (Table 2, Table 3 and Table 4). Similarly, in the tenth and twelfth grade models, reported in Table 3 and Table 4 respectively, adolescents from racial/ethnic minority groups had lower odds than NHW adolescents of dual use when compared to no use. In the eighth grade model, NHB and Hispanic adolescents had higher odds (aOR = 2.20, 95% CI = 1.24–3.90, aOR = 1.70, 95% CI = 1.09–2.66 respectively; Table 2) while Hispanic twelfth graders (aOR = 0.70, 95% CI = 0.52–0.96; Table 4) had lower odds of exclusive combustible use than NHW adolescents. 

In general, higher parental education had an inverse relationship with exclusive combustible and dual use but not with exclusive e-cigarette use (Table 2, Table 3 and Table 4). Younger adolescents whose highest parental education was a 4 year college degree or more had about half the odds of exclusive combustible (eighth grade: aOR = 0.43, 95% CI = 0.23–0.78; tenth grade: aOR = 0.45, 95% CI = 0.24–0.84; and twelfth grade: aOR = 0.65, 95% CI = 0.50–0.86) and dual use (eighth grade: aOR = 0.45, 95% CI = 0.27–0.74; tenth grade: aOR = 0.51, 95% CI = 0.35–0.74) than those whose highest parental education was less than a high school degree. There was no association between parental education and dual use among twelfth graders (Table 4). However, twelfth graders whose highest parental education was a high school degree or more had higher odds of exclusive e-cigarette use (when compared to no use) than those with less than a high school degree (Table 4).

## 4. Discussion

Our study investigated the sociodemographic patterns of exclusive and dual use of e-cigarettes and combustible tobacco products (cigarettes or cigars) among adolescents between 2017 and 2020. In line with previous evidence [2,3,4,6,12], we found that exclusive e-cigarette use was the most prevalent use pattern across all grades [2,3,4,6], followed by the dual use of e-cigarettes and combustible tobacco, which is the most common dual use combination among adolescents [12]. We also observed important use patterns by sociodemographic characteristics. For example, older male adolescents had higher odds of exclusive combustible or dual use than older female adolescents, which is consistent with previous evidence [4,12], while younger male adolescents had lower odds of exclusive e-cigarette use than younger female adolescents, which differs from earlier studies [4]. Regarding race/ethnicity, relative to NHW respondents, racial/ethnic minority respondents had lower odds of exclusive e-cigarette and dual use across all grades, which is consistent with previous research [2,4,12]. Also, as earlier studies show [12,13], we found that adolescents whose highest parental education was a college degree or more versus less than a high school degree had lower odds of exclusive combustible tobacco product and dual use when compared to no use, but higher odds of exclusive e-cigarette use. 

The consistently high exclusive e-cigarette use prevalence across all grades reflects the rapid increase of e-cigarette use in 2018, following the peak popularity of JUUL products in late 2018 and early 2019 [21]. During that period, JUUL’s sales comprised 70% of the e-cigarette marketplace [21]. A 2017 nationally representative study showed that almost half of all adolescents who tried a nicotine/tobacco product started with e-cigarettes [22] and the increased use of e-cigarettes may be contributing to the persistent pattern of dual use among adolescents [4]. For more context, we need research that examines the frequency of e-cigarette use and its association with dual use among adolescents who used e-cigarettes at least once in the past 30 days within our study period.

Our study examined the exclusive use of e-cigarettes, unlike previous studies, which commonly measure any use of e-cigarettes, which include adolescents who have used e-cigarettes exclusively or with other tobacco products [14,23,24]. Previous studies reported that e-cigarette use was more common among males or comparable across males and females in middle school from 2011–2018 [8,23,24]. Similar studies have shown that dual/poly use of any two tobacco products including e-cigarettes is more common among adolescent males, with little to no reference to the exclusive use of the studied products. Our study, however, shows that the exclusive use of e-cigarettes is more common among female adolescents. Distinguishing exclusive e-cigarette use from multiproduct use is essential since associated risk factors vary by tobacco product [16]. It also helps inform interventions suited for specific tobacco products. Studies have shown that female adolescents are more curious about and susceptible to e-cigarettes, and have an increased urge to use e-cigarettes relative to males [2,4]. Female adolescents are also more likely to initiate tobacco use with non-cigarette products such as e-cigarettes [25]. The role of e-cigarettes as a potential transition tool to combustible tobacco product use means female adolescents may be at future risk of combustible product-related health issues [26,27].

In our study, we observed that the odds of combustible tobacco use, with or without e-cigarettes, are higher among male adolescents when compared to females. Results from studies on cigarette use among middle and high schoolers by sex are mixed [3,8,12]. However, cigar or e-cigarette use are consistently more common among males than females [3,8]. Male high schoolers are more susceptible to cigarette and cigar use, potentially as a result of higher second-hand smoke exposure [28], and are more likely to initiate nicotine/tobacco product use with combustible tobacco products when compared to females [25]. With higher odds of exclusive combustible and dual use, male adolescents are more likely than females to be at risk for nicotine dependence, tobacco-related health issues, and to continue using tobacco into adulthood [29].

Nationally representative studies show that e-cigarette use is more common among NHW adolescents than those from racial/ethnic minoritized groups [2,4], consistent with our findings. NHW adolescents have the highest exposure to e-cigarette marketing [30], which is promoted more in NHW and medium- to high-income neighborhoods [30,31]. They also report higher use of cigarettes [2,4], which may explain the higher odds of dual use relative to other racial/ethnic groups. On the other hand, in our study we observed that younger racial/ethnic minority adolescents reported higher odds of using combustible tobacco relative to NHW adolescents; hence, they remain at risk for poor health outcomes associated with combustible tobacco use into adulthood [2,3,4,32]. For example, a study among adults aged 18 to 44 years, showed that NHB individuals who smoked cigarettes are at a higher risk of nicotine dependence and tobacco-related deaths when compared to NHW individuals [33]. With the continued targeted marketing of tobacco products to racial/ethnic minority populations and persistent disparities in use, constant monitoring, and surveillance of tobacco use patterns by race/ethnicity is justified.

Finally, our results corroborate studies that show that higher parental education is associated with lower exclusive combustible use, multiple nicotine/tobacco product use, and higher exclusive e-cigarette use when compared to lower parental education [12,13,34,35]. Individuals from lower socioeconomic households are generally at higher risk of exclusive cigarette or cigar use, partly due to targeted marketing [29]. Furthermore, adults with lower SES smoke cigarettes more frequently, thus exposing their children to tobacco use and potentially leading to their children using similar products as well [36]. With dual use being the second most common pattern among adolescents, our results show that adolescents from lower SES households face additional risks of tobacco-associated health risks. This increased risk is particularly concerning as lower SES households tend to use combustible tobacco products more heavily [37], report higher lung cancer incidence [38], and have less access to health care or cessation services [39,40] when compared to individuals from higher SES households. Tobacco cessation outreaches such as free cessation treatments (nicotine replacement therapy and telephone counseling) have proven effective in lowering nicotine/tobacco product use among lower SES groups [41,42]. Such targeted interventions should be promoted to reduce the existing inequalities in combustible and dual use by SES.

This study has some limitations. First, to account for small cell sizes, we pooled MTF data from 2017–2020 and were therefore unable to report sociodemographic differences in tobacco use patterns from year to year. However, these combined analyses provide greater statistical power to determine differences in tobacco use patterns across sociodemographic subgroups and to detect whether the associations are statistically significant. Other studies using MTF data have also used this approach of combining data across years [18,19]. Second, this study considered adolescents who had used tobacco products at least once during the past 30 days as current users, rather than using a more stringent definition of use such as 10 or more days in the past 30 days. Several youth tobacco studies (including the MTF) as well as the Centers for Disease Control and Prevention define current tobacco use among youth as one or more days/occasions in the past 30 days [4,5,6,8,18,19]. It recognizes that many youth may be experimenting, but that the experimentation is still important to capture. Finally, we combined other non-Hispanic races, except for whites and Blacks, into one category, limiting our ability to make specific inferences for other racial groups such as non-Hispanic Asian, American Indian/Alaskan Native, or multiracial adolescents.

## 5. Conclusions

Overall, our findings add to the growing literature on sociodemographic patterns of adolescent tobacco use by examining patterns of exclusive and dual use of e-cigarettes and combustible tobacco (cigarettes or cigars) among adolescents by sex, race/ethnicity, and parental education from 2017 to 2020 [2,4,12,43]. Even with an overall decline in cigarette or cigar use, dual use of e-cigarettes with combustible tobacco (cigarettes or cigars) remains prevalent, specifically among older adolescents. Adolescents with lower parental education levels are more likely to use combustible tobacco exclusively or with e-cigarettes than adolescents with higher parental education levels, suggesting adolescents with lower SES remain at increased risk of tobacco-associated health risks. Research should continue to monitor the differential use of combustible tobacco products and e-cigarettes among adolescents, specifically among those who have been traditionally targeted by the tobacco industry, including people from low SES backgrounds or from racial/ethnic minority households, in order to inform ongoing and future interventions or policies. 

## Figures and Tables

**Table 1:** Descriptive statistics of study samples of exclusive and dual use of combustibles (cigarette and cigars) and e-cigarettes: Monitoring the Future Surveys 2017 to 2020.

	8th Grade				10th Grade				12th Grade			
	2017–2020				2017–2020				2017–2020			
	*N*	%	95% CI		*N*	%	95% CI		*N*	%	95% CI	
Total	11,189	100			12,882	100			11,385	100		
Sex												
Female	5982	53.5	52.2	54.8	6870	53.3	51.8	54.8	5986	52.6	50.9	54.3
Male	5207	46.5	45.2	47.8	6012	46.7	45.2	48.2	5399	47.4	45.7	49.1
Race/Ethnicity												
NH White	5570	49.8	46.0	53.5	6628	51.5	47.3	55.6	6152	54.0	49.7	58.4
NH Black	1081	9.7	7.7	11.6	1600	12.4	10.1	14.7	1060	9.3	7.4	11.2
NH Other	1595	14.3	12.4	16.1	1454	11.3	10.0	12.5	1235	10.8	9.3	12.4
Hispanic	2942	26.3	22.8	29.7	3200	24.8	21.4	28.3	2938	25.8	21.6	30.0
Highest Parental Education												
High school degree or less	2739	24.5	21.9	27.0	3360	26.1	23.8	28.4	3276	28.8	25.9	31.6
Some College	1649	14.7	13.8	15.7	2005	15.6	14.5	16.7	2161	19.0	17.7	20.2
4 years of college or more	6801	60.8	57.9	63.6	7517	58.4	55.5	61.2	5948	52.2	49.2	55.3
Tobacco Product Use Patterns												
No use	10,007	89.4	88.5	90.4	10,286	79.8	78.2	81.5	8200	72.0	70.0	74.0
Exclusive combustible (cigarettes or cigars) use	166	1.5	1.2	1.8	316	2.5	2.0	2.9	605	5.3	4.6	6.0
Exclusive e-cigarette use	711	6.4	5.6	7.1	1700	13.2	11.9	14.5	1566	13.8	12.3	15.3
Dual use	304	2.7	2.3	3.2	580	4.5	3.9	5.1	1014	8.9	7.9	9.9

**Table 2:** Multinomial Regression Models of Sociodemographic Factors associated with Exclusive and Dual use of E-cigarettes with Combustibles (cigarettes and cigars) among eighth graders: Monitoring the Future Surveys, 2017 to 2020.

	8th Grade (*N* = 11,189)																	
	Unadjusted									Adjusted (Compared to No Use)								
	Exclusive Combustible (Cigarettes/Cigars) Use			Exclusive E-Cigarette Use			Dual Use			Exclusive Combustible (Cigarettes/Cigars) Use			Exclusive E-Cigarette Use			Dual Use		
	OR	[95% Conf.]		OR	[95% Conf.]		OR	[95% Conf.]		OR	[95% Conf.]		OR	[95% Conf.]		OR	[95% Conf.]	
Sex																		
Female	(Referent)																	
Male	1.06	0.75	1.50	0.80 *	0.67	0.96	1.26	0.94	1.69	1.09	0.77	1.54	0.81 *	0.68	0.98	1.30	0.97	1.76
Race/Ethnicity																		
NH White	(Referent)																	
NH Black	2.48 ***	1.40	4.40	0.35	0.22	0.55	0.75	0.45	1.27	2.20 *	1.24	3.90	0.33 ***	0.21	0.53	0.69	0.40	1.19
NH Other	2.10	0.94	4.66	0.65 *	0.46	0.91	1.24	0.72	2.14	2.00	0.91	4.40	0.63 *	0.45	0.88	1.20	0.69	2.10
Hispanic	2.23 ***	1.48	3.36	0.89	0.71	1.12	1.80 ***	1.32	2.45	1.70 *	1.09	2.66	0.81	0.64	1.02	1.41	0.99	2.03
Highest Parental Education																		
High school degree or less	(Referent)																	
Some college	0.67	0.39	1.17	1.34 *	1.01	1.78	0.74	0.51	1.09	0.73	0.42	1.27	1.28	0.96	1.71	0.79	0.53	1.18
4 years of college or more	0.43 ***	0.28	0.65	0.85	0.67	1.08	0.48 ***	0.24	0.69	0.49 **	0.31	0.76	0.80	0.62	1.03	0.54 **	0.37	0.80
Year																		
2017	(Referent)																	
2018	0.72	0.45	1.14	2.09 ***	1.62	2.69	1.07	0.75	1.53	0.72	0.46	1.13	2.09 ***	1.62	2.70	1.09	0.77	1.55
2019	1.03	0.62	1.71	1.60 ***	1.23	2.09	0.86	0.57	1.30	1.05	0.64	1.73	1.66 ***	1.28	2.17	0.92	0.61	1.39
2020	0.69	0.32	1.50	1.52	0.97	2.38	1.08	0.50	2.36	0.74	0.33	1.67	1.50	0.96	2.35	1.15	0.51	2.57

* *p* < 0.05; ** *p* < 0.01; *** *p* < 0.001.

**Table 3:** Multinomial Regression Models of Sociodemographic Factors associated with Exclusive and Dual use of E-cigarettes with Combustibles (cigarettes and cigars) among tenth graders: Monitoring the Future Surveys, 2017 to 2020.

	10th Grade (*N* = 12.882)																	
	Unadjusted									Adjusted								
	Exclusive Combustible (Cigarettes/Cigars) Use			Exclusive E-Cigarette Use			Dual Use			Exclusive Combustible (Cigarettes/Cigars) Use			Exclusive E-Cigarette Use			Dual Use		
	OR	[95% Conf.]		OR	[95% Conf.]		OR	[95% Conf.]		OR	[95% Conf.]		OR	[95% Conf.]		OR	[95% Conf.]	
Sex																		
Female	(Referent)																	
Male	0.99	0.70	1.41	0.95	0.82	1.10	1.68 ***	1.34	2.10	1.02	0.72	1.45	0.93	0.81	1.08	1.67 ***	1.33	2.09
Race/Ethnicity																		
NH White	(Referent)																	
NH Black	1.44	0.95	2.18	0.27 ***	0.20	0.37	0.20 ***	0.12	0.33	1.27	0.84	1.91	0.27 ***	0.19	0.37	0.19 ***	0.11	0.31
NH Other	1.30	0.79	2.14	0.65 ***	0.51	0.82	0.69	0.48	0.99	1.27	0.76	2.13	0.65 ***	0.50	0.83	0.69 *	0.48	0.99
Hispanic	1.23	0.75	2.01	0.62 ***	0.52	0.76	0.69	0.45	1.05	0.95	0.54	1.70	0.59 ***	0.49	0.71	0.56 **	0.37	0.85
Highest Parental Education																		
High school degree or less	(Referent)																	
Some college	0.71	0.43	1.16	1.17	0.95	1.43	0.96	0.73	1.27	0.71	0.44	1.14	1.05	0.87	1.28	0.88	0.66	1.16
4 years of college or more	0.46 ***	0.33	0.65	1.13	0.95	1.43	0.73 **	0.57	0.92	0.46 *	0.32	0.64	0.96	0.81	1.13	0.59 ***	0.46	0.74
Year																		
2017	(Referent)																	
2018	0.99	0.60	1.66	2.77 ***	2.17	3.53	1.46 *	1.08	1.98	1.00	0.61	1.64	2.77 ***	2.19	3.51	1.46 *	1.08	1.97
2019	0.55 *	0.35	0.84	2.99 ***	2.25	3.98	1.16	0.78	1.73	0.55 *	0.36	0.84	3.05 ***	2.34	3.97	1.19	0.81	1.75
2020	0.45 *	0.24	0.81	2.07 ***	1.46	2.93	0.67	0.41	1.10	0.45 *	0.25	0.83	2.05 ***	1.47	2.86	0.69	0.43	1.11

* *p* < 0.05; ** *p* < 0.01; *** *p* < 0.001.

**Table 4:** Multinomial Regression Models of Sociodemographic Factors associated with Exclusive and Dual use of E-cigarettes with Combustibles (cigarettes and cigars) among twelfth graders: Monitoring the Future Surveys, 2017 to 2020.

	12th Grade (*N* = 11,385)																	
	Unadjusted									Adjusted								
	Exclusive Combustible (Cigarettes/Cigars) Use			Exclusive E-Cigarette Use			Dual Use			Exclusive Combustible (Cigarettes/Cigars) Use			Exclusive E-Cigarette Use			Dual Use		
	OR	[95% Conf.]		OR	[95% Conf.]		OR	[95% Conf.]		OR	[95% Conf.]		OR	[95% Conf.]		OR	[95% Conf.]	
Sex																		
Female	(Referent)																	
Male	1.46 ***	1.18	1.81	1.12	0.95	1.31	2.21 ***	1.86	2.64	1.49 ***	1.21	1.84	1.08	0.92	1.27	2.16 ***	1.82	2.57
Race/Ethnicity																		
NH White	(Referent)																	
NH Black	0.77	0.52	1.14	0.22 ***	0.15	0.31	0.19 ***	0.11	0.32	0.73	0.49	1.08	0.23 ***	0.16	0.33	0.19 ***	0.12	0.32
NH Other	0.84	0.53	1.31	0.62 ***	0.49	0.78	0.54 **	0.38	0.77	0.81	0.53	1.25	0.63 ***	0.50	0.79	0.55 **	0.39	0.77
Hispanic	0.78	0.58	1.04	0.46 ***	0.37	0.58	0.41 ***	0.30	0.55	0.70 *	0.52	0.96	0.48 ***	0.38	0.61	0.39 ***	0.29	0.53
Highest Parental Education																		
High school degree or less	(Referent)																	
Some college	0.97	0.70	1.34	1.37 **	1.10	1.72	0.97	0.74	1.28	0.88	0.63	1.22	1.22	0.98	1.52	0.81	0.62	1.07
4 years College+	0.76 *	0.59	0.97	1.60 ***	1.31	1.95	1.23	0.96	1.57	0.65 **	0.50	0.86	1.27 *	1.05	1.53	0.89	0.70	1.13
Year																		
2017	(Referent)																	
2018	0.68 *	0.51	0.91	2.67 ***	2.06	3.46	1.23	0.95	1.60	0.68 **	0.51	0.91	2.67 ***	2.10	3.38	1.22	0.97	1.55
2019	0.42 ***	0.29	0.59	3.11 ***	2.38	4.07	1.14	0.85	1.54	0.41 ***	0.29	0.59	3.12 ***	2.46	3.94	1.13	0.86	1.49
2020	0.33 ***	0.18	0.60	2.92 ***	1.97	4.33	1.29	0.68	2.43	0.32 ***	0.18	0.59	3.17 ***	2.28	4.41	1.34	0.72	2.48

* *p* < 0.05; ** *p* < 0.01; *** *p* < 0.001.

## Data Availability

Restricted Monitoring the Future Panel Data can be accessed at www.icpsr.umich.edu (accessed on 1 November 2020), though permission and approval are needed to access restricted data files.

## Supplementary Materials

The following supporting information can be downloaded at: https://www.mdpi.com/article/10.3390/ijerph19052965/s1, Supplemental Table S1: Monitoring the Future 2017–2020 Selected Forms and Questions.
