# Supplementary material for: Sociodemographic Patterns of Exclusive and Dual Combustible Tobacco and E-Cigarette Use among US Adolescents—A Nationally Representative Study (2017–2020)
Source: Int J Environ Res Public Health. 2022 Mar 3;19(5):2965. doi: 10.3390/ijerph19052965 (PMC8910207; doi:10.3390/ijerph19052965)
Supplement: Supplementary file 1 [file ijerph-19-02965-s001.zip › ijerph-1563654-supplementary.pdf]

Supplemental Table S1: Monitoring the Future 2017-2019 Selected Forms and Questions

**8th & 10th grade**				
		2017	2018	2019
Cigarettes	Form 2, 3&4	"How frequently have you smoked cigarettes during the past 30 days?	"How frequently have you smoked cigarettes during the past 30 days?	"How frequently have you smoked cigarettes during the past 30 days?
E-cigarettes	Form 2	During the LAST 30 DAYS, on how many days (if any) have you used an electronic vaporizer such as an e-cigarette?	On how many DAYS (if any) have you vaped NICOTINE… c) in the past 30 days?	On how many days (if any) have you vaped NICOTINE…in the last 30 days
	Form 3&4	During the LAST 30 DAYS, on how many days (if any) have you…vaped an e-liquid with nicotine?	On how many DAYS (if any) have you used an e-cigarette? c) in the past 30 days?	
Cigars	Form 2	During the LAST 30 DAYS, on how many days (if any) have you…smoked flavored little cigars or cigarillos?<br><br>During the LAST 30 DAYS, on how many days (if any) have you…smoked regular little cigars or cigarillos?	During the LAST 30 DAYS, on how many days (if any) have you…smoked flavored little cigars or cigarillos?<br><br>During the LAST 30 DAYS, on how many days (if any) have you…smoked regular little cigars or cigarillos?	During the LAST 30 DAYS, on how many days (if any) have you…smoked flavored little cigars or cigarillos?<br><br>During the LAST 30 DAYS, on how many days (if any) have you…smoked regular little cigars or cigarillos?<br><br>During the LAST 30 DAYS, on how many days (if any) have you…smoked large cigars?
	Form 3	During the LAST 30 DAYS, on how many days (if any) have you…smoked flavored little cigars or cigarillos?<br><br>During the LAST 30 DAYS, on how many days (if any) have you…smoked regular little cigars or cigarillos?<br><br>During the LAST 30 DAYS, on how many days (if any) have you…smoked large cigars?	During the LAST 30 DAYS, on how many days (if any) have you…smoked flavored little cigars or cigarillos?<br><br>During the LAST 30 DAYS, on how many days (if any) have you…smoked regular little cigars or cigarillos?<br><br>During the LAST 30 DAYS, on how many days (if any) have you…smoked large cigars?	During the LAST 30 DAYS, on how many days (if any) have you…smoked flavored little cigars or cigarillos?<br><br>During the LAST 30 DAYS, on how many days (if any) have you…smoked regular little cigars or cigarillos?<br><br>During the LAST 30 DAYS, on how many days (if any) have you…smoked large cigars?
	Form 4		During the LAST 30 DAYS, on how many days (if any) have you…smoked large cigars?	During the LAST 30 DAYS, on how many days (if any) have you…smoked large cigars?
**12th grade**				
		2017	2018	2019
Cigarettes	Form 2	"How frequently have you smoked cigarettes during the past 30 days?	"How frequently have you smoked cigarettes during the past 30 days?	"How frequently have you smoked cigarettes during the past 30 days?
	Form 4	"How frequently have you smoked cigarettes during the past 30 days?	"How frequently have you smoked cigarettes during the past 30 days?	"How frequently have you smoked cigarettes during the past 30 days?
	Form 5	"How frequently have you smoked cigarettes during the past 30 days?	"How frequently have you smoked cigarettes during the past 30 days?	
E-cigarettes	Form 2	On how many days (if any) have you vaped NICOTINE…during the last 30 days	On how many days (if any) have you vaped NICOTINE…during the last 30 days	On how many days (if any) have you vaped NICOTINE…during the last 30 days
	Form 4	On how many days (if any) have you vaped NICOTINE…during the last 30 days	On how many days (if any) have you vaped NICOTINE…during the last 30 days	On how many days (if any) have you vaped NICOTINE…during the last 30 days
	Form 5	On how many occasions (if any) have you vaped NICOTINE…during the last 30 days	On how many occasions (if any) have you vaped NICOTINE…during the last 30 days	
Cigars	Form 2	During the LAST 30 DAYS, on how many days (if any) have you…smoked flavored little cigars or cigarillos?	During the LAST 30 DAYS, on how many days (if any) have you…smoked flavored little cigars or cigarillos?	During the LAST 30 DAYS, on how many days (if any) have you…smoked flavored little cigars or cigarillos?

		During the LAST 30 DAYS, on how many days (if any) have you…smoked regular little cigars or cigarillos?<br><br>During the LAST 30 DAYS, on how many days (if any) have you…smoked large cigars?	During the LAST 30 DAYS, on how many days (if any) have you…smoked regular little cigars or cigarillos?<br><br>During the LAST 30 DAYS, on how many days (if any) have you…smoked large cigars?	During the LAST 30 DAYS, on how many days (if any) have you…smoked regular little cigars or cigarillos?<br><br>During the LAST 30 DAYS, on how many days (if any) have you…smoked large cigars?
	Form 4	During the LAST 30 DAYS, on how many days (if any) have you…smoked flavored little cigars or cigarillos?<br><br>During the LAST 30 DAYS, on how many days (if any) have you…smoked regular little cigars or cigarillos?	During the LAST 30 DAYS, on how many days (if any) have you…smoked flavored little cigars or cigarillos?<br><br>During the LAST 30 DAYS, on how many days (if any) have you…smoked regular little cigars or cigarillos?	During the LAST 30 DAYS, on how many days (if any) have you…smoked flavored little cigars or cigarillos?<br><br>During the LAST 30 DAYS, on how many days (if any) have you…smoked regular little cigars or cigarillos?<br><br>During the LAST 30 DAYS, on how many days (if any) have you…smoked large cigars?
	Form 5	During the LAST 30 DAYS, on how many days (if any) have you…smoked large cigars?	During the LAST 30 DAYS, on how many days (if any) have you…smoked large cigars?	During the LAST 30 DAYS, on how many days (if any) have you…smoked large cigars?
